# Detecting the “invisible fraction” bias in resurrection experiments

**DOI:** 10.1111/eva.12533

**Published:** 2017-09-23

**Authors:** Arthur E. Weis

**Affiliations:** ^1^ Department of Ecology and Evolutionary Biology University of Toronto Toronto ON Canada; ^2^ Koffler Scientific Reserve at Jokers Hill University of Toronto King City ON Canada

**Keywords:** climate change, experimental evolution, project baseline, rapid evolution

## Abstract

The resurrection approach is a powerful tool for estimating phenotypic evolution in response to global change. Ancestral generations, revived from dormant propagules, are grown side by side with descendent generations in the same environment. Phenotypic differences between the generations can be attributed to genetic change over time. Project Baseline was established to capitalize on this potential in flowering plants. Project participants collected, froze, and stored seed from 10 or more natural populations of 61 North American plant species. These will be made available in the future for resurrection experiments. One problem with this approach can arise if nonrandom mortality during storage biases the estimate of ancestral mean phenotype, which in turn would bias the estimate of evolutionary change. This bias—known as the “invisible fraction” problem—can arise if seed traits that affect survival during storage and revival are genetically correlated to postemergence traits of interest. The bias is trivial if seed survival is high. Here, I show that with low seed survival, bias can be either trivial or catastrophic. Serious bias arises when (i) most seeds deaths are selective with regard to the seed traits, and (ii) the genetic correlations between the seed and postemergence traits are strong. An invisible fraction bias can be diagnosed in seed collections that are family structured. A correlation between the family mean survival rate and the family mean of a focal postemergence trait indicates that seed mortality was not random with respect to genes affecting the focal trait, biasing the sample mean. Fortunately, family structure was incorporated into the sampling scheme for the Project Baseline collection, which will allow bias detection. New and developing statistical procedures that can incorporate genealogical information into the analysis of resurrection experiments may enable bias correction.

## 1 INTRODUCTION

Global change, including shifting land use, species translocations among continents, rising atmospheric CO_2_, and warming climate (Vitousek, 1994), will likely drive evolutionary change in many species during this century (Davis & Shaw, 2001; Thomas et al., 2001; Franks & Hoffmann, 2012). Several adaptive evolutionary responses to anthropogenic change have already been documented (e.g., Carroll, Klassen, & Dingle, 1998; Réale, McAdam, Boutin, & Berteaux, 2003; Levitan & Etges, 2005; Colautti & Barrett, 2013).

To date, serendipity has ruled in studies detecting evolutionary response to climate shift. Peter and Rosemary Grant were studying bird ecology when by chance a severe drought altered food resources, spurring evolution of the finch's beak (Grant & Grant, 2002). Umina, Weeks, Kearney, McKechnie, and Hoffmann (2005) could demonstrate allelic shifts in loci associated with *Drosophila* thermal tolerance, and Bradshaw and Holzapfel (2008) could show a shortening of the critical photoperiod for mosquito diapause, because these research groups had solidly established the clinal variation in these traits more than a decade before climate change became a research focus. Steven Franks, Shiena Sims, and I showed accelerated flowering time in field mustard (*Brassica rapa*) after prolonged drought, using the “resurrection paradigm” (Franks, Sim, & Weis, 2007). This experiment was possible only because several years prior to the drought, Denise Franke Kind (Franke et al., 2006) fortuitously stored the excess seed she collected (for an unrelated project) under conditions that preserved viability.

*Project Baseline* (Franks et al., 2008; Franks, Hamann, & Weis, 2017; Etterson et al., 2016) was established to move the exploration of plant evolutionary response to global change beyond serendipity. This collaborative effort has collected and stored seed from contemporary populations of 61 species. One to two‐hundred maternal sibships have been collected from each of 10 or more populations per species across their geographic ranges. These seeds are being stored in conditions expected to maintain their viability well into the second half of this century. This collection and storage phase was implemented between 2013 and 2016. The payoff for this initial effort will come only in future years. Researchers who collect seed from the same populations can withdraw ancestral seed from the collection to do resurrection experiments.

The resurrection approach is a very powerful way to study phenotypic evolution. Dormant propagules produced by an ancestral generation are revived and grown side by side with propagules from a more recent generation. Because both generations develop in the same environment, phenotypic differences between them can be attributed to evolutionary (genetic) change over time. Richard Lenski and associates have applied it in their ongoing evolution experiment with *Escherichia coli,* where they have measured evolutionary rates, uncovered the physiological basis of adaptation, and examined the contingency of evolutionary trajectories (Lenski, Rose, Simpson, & Tadler, 1991; Elena, Cooper, & Lenski, 1996; Blount, Borland, & Lenski, 2008).

Resurrection experiments have documented adaptive evolution in natural populations as well. Using *Daphnia galeata* hatched from resting eggs retrieved from sediments in Lake Constance, Hairston et al. (1999) found an increase, followed by a decrease, in resistance to toxic cyanobacteria between the 1960s and 1990s, matching the rise and fall of lake eutrophication. Frisch et al. (2014) examined adaptation to eutrophication by *Daphnia pulicaria* using clones resurrected from sediments as old as 700 years. Changes in *Daphnia* behavior in response to shifting predation risk have also been detected by comparing contemporary to resurrected clones (Cousyn et al., 2001). Plants too have been the subject of several recent resurrection experiments (Bustos‐Segura, Fornoni, & Nunez‐Farfan, 2014; Nevo et al., 2012; Sultan, Horgan‐Kobelski, Nichols, Riggs, & Waples, 2013; Thomann, Imbert, Engstrand, & Cheptou, 2015). With the first release of Project Baseline seeds scheduled for 2018, it is important to consider potential limitations and pitfalls to experiments that attempt to revive ancestral genotypes.

### 1.1 Confronting caveats

Though powerful, the resurrection approach is subject to three biases (Bennington & McGraw, 1995). The first is caused when differences in the collection protocol for the two generations select different sets of genotypes. For example, ancestral generation seeds collected from the wet side of the meadow may be genetically different from descendants collected from the dry side, but that difference could be entirely due to local adaptation at the microgeographic level and not due to evolutionary change across time. Similarly, differences in the timing of collection relative to seed maturation could distort the true level of genetic change in phenology between generations. Consideration of these issues in the sampling design can ameliorate this concern (see Franks et al., 2017).

A second bias can arise when propagules from the ancestral and descendent generations have been produced and stored under different conditions, triggering plastic responses in postemergence phenotypes (Rogalski, 2015). These effects can be ameliorated by rearing the propagules in a common environment for one or more generations before estimating phenotypic divergence (e.g., Hairston et al., 1999). These “refresher generations” can also be used to produce intergenerational hybrids, which can reveal genetic features of change (Franks et al., 2007). Several refresher generations may be required to eliminate epigenetic modifications. Great care must be taken to avoid unintentional selection during the refresher generations. Breeding protocols that ensure equal contributions of all individuals, male and female, to each subsequent generation can all but eliminate the opportunity for selection. When study species have long generation times, refresher generations can be impractical, and so results need to be interpreted with caution.

In a similar vein, loss of symbionts during storage could lead to phenotypic differences between ancestors and descendants. Cheplick (2017) found 25% to 40% of *Lolium perenne* seeds revived after 22 years of storage no longer harbored the beneficial endophytic fungi are typically inherited maternally. In such a case, re‐infection during a refresher generation would be indicated for a fair test of evolutionary change.

This article will focus on the third potential bias in resurrection experiments that arising from nonrandom mortality of ancestors during storage (Bennington & McGraw 1995). Suppose a variable trait expressed in the propagule affects its chance of surviving prolonged storage. If that propagule trait is genetically correlated to a focal postemergence trait (e.g., growth rate, specific leaf area, flowering date), the genotypes emerging from storage will be a nonrandom sample of those that went in. This will bias the estimated ancestral mean of the focal trait, which is the baseline for estimating evolutionary change.

Grafen (1988) called this the problem of the “invisible fraction”. Net selection on loci underlying a late‐life trait will be under/overestimated if effects of those loci on early‐life survival are not taken into account (Bennington & McGraw, 1995; Brommer, Merilä, & Kokko, 2002). Similar biases arise in estimates of quantitative genetic variances and covariances if no accounting is made for the invisible fraction (Hadfield, 2008; Nakagawa & Freckleton, 2008; Kruuk, Slate, & Wilson, 2008).

Genetic variation in the ability to survive storage could lead to an invisible fraction bias when resurrecting the Project Baseline collection. Accessions for the 61 species have been dried to 20% RH and stored at −20°C at the National Center for Genetic Resources Preservation (Etterson et al., 2016). These are the conventional storage conditions used by gene banks for preserving agronomic species, and they are expected to maintain seed viability for 50 to 400 years (FAO 2013). Some wild species, however, have poorer desiccation and/or freezing tolerance than cultivars (Walters, 2015), and so their longevity under storage could be shorter. The 61 target species for Project Baseline were identified as good candidates for storage. But, what if upon resurrection the germination rate among the stored ancestors is lower than the freshly collected descendants? How strongly could this bias the baseline for estimating phenotypic evolution? How do we detect bias? After placing the issue within the framework of missing data theory, I examine the potential magnitude of the invisible fraction bias in resurrection experiments and offer approaches to detect and account for it.

### 1.2 Conceptual background

The invisible fraction problem is a missing data problem (Hadfield, 2008). When stored seeds fail to germinate, or dormant eggs perish in the sediment before collection (Hairston, Van Brunt, Kearns, & Engstrom, 1995), data on their postemergence phenotype are missing from the analysis. If emergence rate is high, few data are missing and so bias in estimates of the ancestral phenotypic mean is likely negligible. Low emergence rates can lead to strong bias, but not necessarily. It depends whether the genetic correlation between failure and focal phenotype is strong or weak.

**Figure 1:**
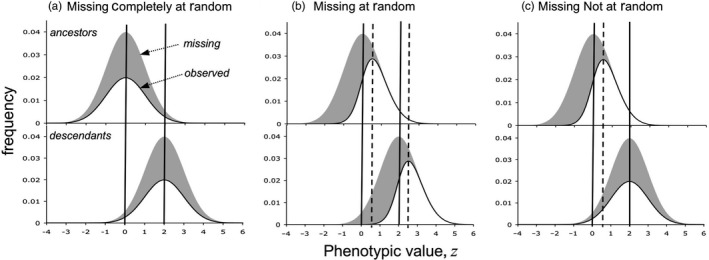
Contrasting the phenotypic distribution of trait *z* in ancestral and descendant generations of a resurrection experiment. Shaded portions of the Gaussian distributions represent the potential phenotypes of individuals missing because of early mortality (the missing fraction), while the unshaded portions are phenotypes of individuals that survived to have the trait measured. (a) In both generations, the factor causing early mortality acts independently of the potential/realized value of *z*; data for the dead individuals are missing at random. The difference between generation means is the same as if all had survived and so is estimated without bias. (b) In both generations, the factor causing early mortality declines with *z*, but it does so equally within each generation. The two means are estimated with equal bias such that the difference between them is estimated without bias. (c) The factors causing early mortality depend unequally on *z* between generations. The ancestral and descendant generation means are estimated with unequal biases, and so the difference between them will over/under estimate the expected difference had all survived

In the parlance of missing data theory, bias occurs when the process under study in some way depends on the process generating missingness (Little & Rubin, 2002). In this view, there are three ways in which data can be missing. First, data can be Missing Completely at Random (MCAR), meaning complete independence between the two processes. Within the context of a resurrection experiment, factors causing emergence failure are the same across all genotypes in both generations (Figure 1a) and unrelated to postemergence phenotype. Even if emergence rates differ between ancestors and descendants, the evolutionary shift in phenotypic mean is estimated without bias in the MCAR case. Second, data may be Missing at Random (MAR). Here, the process generating missingness may affect response variables, but in the context of a resurrection experiment, its relative effect does not vary across generations. As indicated in Figure 1b, propagules that would produce low ranking values for the focal phenotype are less likely to emerge, but because failure is tied to rank within the generation, and not rank across generations, the estimate for evolutionary shift in phenotypic mean is unbiased. This holds so long as germination rates are the same in the two generations. The missingness of data that are MCAR and MAR can be ignored when making inferences on the process of interest (Little & Rubin, 2002).

Data Missing Not at Random (MNAR) cannot be ignored. Here, the process generating missingness changes in intensity and/or direction with the process under study (Figure 1c). With regard to a resurrection experiment, imagine a propagule trait that affects the chances of emergence after decades of storage. If that propagule trait is genetically correlated with a focal trait expressed postemergence, ancestors and descendants will appear to have diverged, even if selection imposed by global change—the process of interest—is zero.

## 2 THE POTENTIAL MAGNITUDE OF THE MISSING FRACTION BIAS

To illustrate the potential invisible fraction bias, suppose the following. We are interested in some focal trait *z* that is expressed in mature plants. A very large, random sample of seeds was collected from a natural population, dried, frozen, and then revived at a later date. These are grown side by side with a descendent generation. All descendent seeds germinate, but fraction *q* of the ancestors fails to revive. Back at the time of collection (time 0), the potential mean value for focal trait *z* within the ancestral sample was ${\overset{¯}{z}}_{0}$, which is the proper baseline for testing evolutionary change in *z*. However, *z* is expressed at time *t*, after revival and emergence, when only 1‐*q* of the ancestral sample remains. Finally, assume that failure depends on seed trait *y*. To simplify calculations, assume *z* and *y* are normally distributed with zero mean and unit variance.

How much will the phenotypic mean of the germinated ancestors, ${\overset{¯}{z}}_{t}$_,_ differ from ${\overset{¯}{z}}_{0}$? Drawing on basic quantitative genetic theory (Lynch & Walsh, 1998), the bias can be stated as

(1) $${\overset{¯}{z}}_{t}-{\overset{¯}{z}}_{0}=G_{zy}\beta_{y}$$

where *G*_*zy*_ is the genetic covariance between the seed and focal traits, and β_*y*_ is the selection gradient acting on *y* through storage survival. Absolute selection intensity, |β_*y*_|, will increase with mortality, *q*, in some fashion, and, with it, the potential for bias. No terms exclusively for *z* appear on the right‐hand side because the events quantified by Equation (1) occur before *z* is expressed. Clearly, if *G*_*zy*_ = 0, the data missing from the estimate of ${\bar{z}}_{t}$ are MCAR, and the baseline is unbiased by the storage process.

Equation (1) can illustrate the relationship of bias to mortality rate when *G*_*zy*_ ≠ 0. Truncation selection is the most extreme way for *q* to relate to β_*y*_ (Crow & Kimura, 1979). All seeds with trait values below threshold y* fail, while those with values above the threshold germinate. In this case, *q* is the area under the normal curve below y*. The standard quantitative genetic formulation for the intensity for truncation selection, when variance is 1.0, yields the following selection gradient: β_*y*_ = *p*(y*)/(1–*q*), where *p*(y*) is the probability of drawing an individual measuring below y* from a normal distribution.

**Figure 2:**
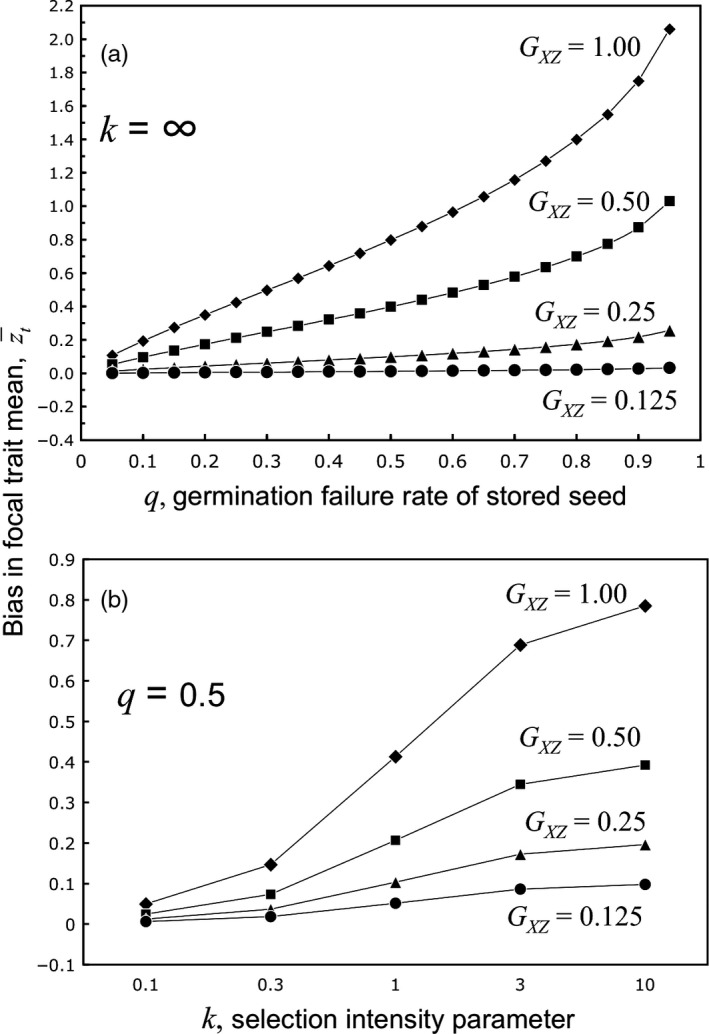
Bias in the estimated ancestral mean phenotype increases with the germination failure (mortality) rate and with the stringency of selection on seed traits affecting germination failure. (a) Under truncation selection (*k* = ∞), bias increases strongly with germination failure rate when the seed trait *y* is perfectly genetically correlated with the adult trait *z* (*G*_*zy*_^ ^= 1.0), but bias is negligible when the correlation is weak. (b) At a given germination failure rate, bias can be strong when the failure is overwhelmingly determined by trait *y* (*k *=* *100), but the strength of the relationship depends on *G*_*zy*_. If additional factors unrelated to *y* are the overwhelming cause of mortality (*k* << 1.0), bias is weak even if *G*_*zy*_ is large

Figure 2a shows that with truncation selection, ${\bar{z}}_{t}$ can deviate dramatically from ${\bar{z}}_{0}$ when mortality is high and the genetic covariance is strong. Under a “worst‐case scenario” (*q *=* *0.95, *G*_*xy*_ = 1), the baseline to measure evolutionary change is off by 2 *SD*s. However, the bias is very weak (~0.01 *SD*) when mortality is only 5% and the genetic correlation is 0.25. At moderate mortality rates and genetic correlations, truncation selection can still distort the baseline by 0.1 to 0.4 *SD*s (Figure 2a), which is sufficient to obscure (or inflate) a biologically significant evolutionary response.

Calculations based on truncation selection set an upper boundary on potential bias for a given mortality rate. A weaker relationship of mortality to selection ameliorates the impact of a high genetic correlation. Suppose the overall failure rate is 0.5, but the chance that a given seed germinates is a logistic function of trait *y*:

(2) $$W\;=\;\frac{1}{1\;+\;e^{ky}}$$

where *W* is the survival component of absolute fitness through the storage/revival selection episode. The stringency parameter of this fitness function, *k*, determines how abruptly failure increases with *y*. Figure 1S shows that at *k *=* *10, selection is approximately truncating; nearly, all seeds with *y* values below the mean fail, while those above succeed. When *k *=* *0.1, the chance of failure still increases with *y*, but most failures are independent of seed trait *y*. In both cases, *q *=* *0.5, but the selection gradient on *y* falls from ~0.80 when *k *=* *10, to ~0.05 when *k *=* *0.1. Thus, seed mortality by itself is a poor predictor of bias. Holding *q* at 0.5, Figure 2b shows that a weak slope to the fitness function (low *k*) generates low bias, even under strong genetic correlations.

To repeat, if seed germination failure is very low, bias will be trivial. But the bias under high failure rates can be either trivial or catastrophic, depending on how strongly mortality genetically correlates with the focal trait. One cannot determine the strength of this correlation from an unstructured sample of propagules. However, if genealogical relationships within the ancestral sample are known, the potential postemergence phenotype of a failed propagule can be inferred from phenotypes of its surviving relatives (Hadfield, 2008; Steinsland, Larsen, Roulin, & Jensen, 2014). The next section shows how relatedness among individuals can be used to detect and account for an invisible fraction problem.

## 3 DETECTING AND ACCOUNTING FOR THE INVISIBLE FRACTION

**Figure 3:**
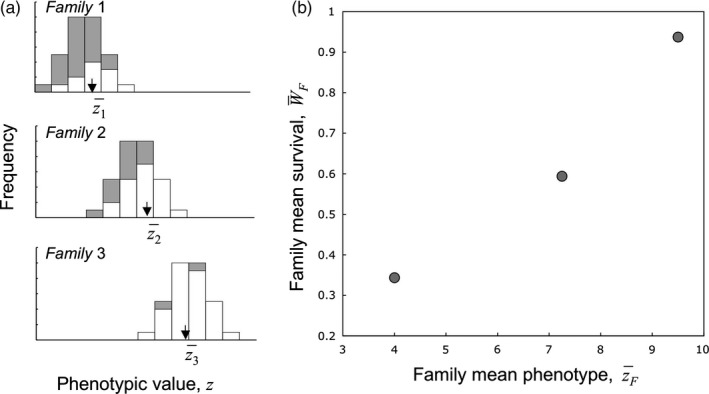
When seed trait *y*, which causes mortality, is strongly correlated with adult trait *z*, families with high values of *z* will also have survival rates. (a) The distribution of potential z values for all siblings, missing (shaded bars) and measured (open bars; see Figure 1). (b) The correlation between the family means for *z* and the proportion of siblings surviving to express *z*

An invisible fraction bias can be detected when data on juvenile survival and later‐life phenotypes are collected for distinct genotypic classes. Mojica and Kelly (2010) presented a salient example in a 3‐year field experiment on the wildflower *Mimulus guttatus*. Previous studies had consistently shown that plants with wider corollas produced more seed, signifying upward selection on flower size. Because these studies examined only mature plants from wild populations, no information could be retrieved on the relationship of flower size to juvenile survival—the plants that died young were missing from the sample used to estimate selection intensity. Mojica and Kelly's study used plants from an artificial selection experiment: seedlings from large‐selected, small‐selected, and control lines for corolla width were planted into the field. Considering only the plants that survived to adulthood, the line selected for larger flowers had a twofold advantage in seed production over the small‐flowered line—a result concordant with previous studies. However, not all plants survived to flowering. Juvenile mortality for the large‐flowered line was 10 times greater than for the small. Clearly, the mean flower size among all survivors was smaller than it would have been if all plants from the large‐flowered line had survived to maturity. Even though flower sizes for the dead plants were missing, the resulting bias could be detected because each deceased plant could be assigned to either the large‐ or small‐flowered line. Phrased differently, the potential phenotype of the missing individuals could be inferred from the phenotypes of their surviving relatives (Figure 3).

**Figure 4:**
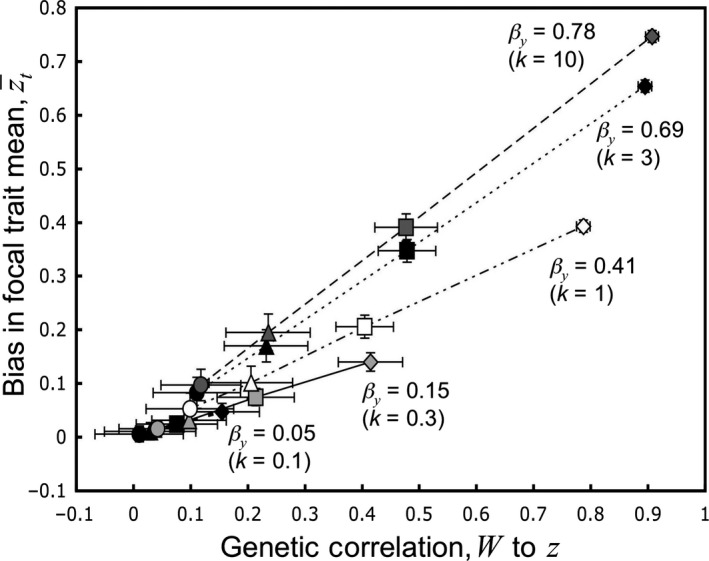
Bias in estimates of the phenotypic mean for z increases with the genetic correlation between *z* and probability of survival, *W*. Each series in the graph represents a different level of stringency in the relation of seed trait *y* to survival, with *k *=* *100 indicating that *y* has overwhelming influence on *W*, whereas *k *=* *0.1 indicates that other factors overwhelmingly influence survival. Symbols for the series are varied in shading to increase clarity. The points in each series, from left to right, indicate increasing levels of genetic correlation between *y* and *z* (circles, *G*_*zy*_ **= 0.125; triangles, *G*_*zy*_ = 0.25; squares, *G*_*zy*_ = 0.50; diamonds, *G*_*zy*_ = 1.0). If *y* determines survival, *W*, and if *y* is strongly correlated to *z*, then the correlation between *W* and *z* will likewise be strong. When *y* has scant influence on *W*, the genetic correlation between *W* and *z* will be weak, no matter what the genetic correlation between *y* and *z*. Overall, a strong genetic correlation of *W* to *z* indicates that the missing fraction problem is causing a strong bias in the estimate of the phenotypic mean of *z*

Returning to the hypothetical situation from the previous section, quantitative genetic methods can detect genetic correlations between seed traits affecting storage mortality and a later‐expressed plant trait *z*. Although the seed traits are unknown and hence unmeasured, they can be correlated with the survival fitness component, *W*, which can be known for each seed in a properly designed experiment. If *z* is genetically correlated to hypothetical *y*, and *y* is correlated to *W*, then there will be a genetic correlation between *z* and *W*. Figure 4 illustrates a situation in which families with low values of *z* have low storage survivorship, while high *z* families have high. The inference from the genealogical relationships among plants is that alleles increasing *z* also increase traits promoting seed survival.

To illustrate the relationship of bias in ${\bar{z}}_{t}$ to *G*_*W,z*_, I ran simulations incorporating the same family structure as the Project Baseline collections. This genetic correlation was approximated from the correlation of the family means for *z* and *W*. R code for the simulation is found in the Data S1. Figure 4 shows the mean bias for simulations of a resurrected baseline generation comprised of 200 half‐sib families with 20 sibs per family, where the population mean mortality was *q *=* *0.5. One hundred simulations were run for each of the *k*/*G*_*zy*_ combinations illustrated in Figure 2b. The simulation shows that a moderate to strong genetic correlation between *W* and *z* clearly indicates a biased estimate of ${\bar{z}}_{t}$ . When *G*_*W,z *_< 0.2, the bias tends to be 0.1 *SD* or less. I emphasize, however, that while *G*_*W,z*_ can be used to flag a serious invisible fraction problem, it does not by itself correct the bias.

Recently, Steinsland et al. (2014) offered an approach to deal with the missing fraction problem that uses a shared parameter model (SPM; Vonesh, Greene, & Schluchter, 2006), implemented in a Bayesian framework. These models assume a conditional independence between the data model and a model for the missingness process. Steinsland et al. (2014) should be consulted for details, but a brief description follows. Consider vector **z**, which contains the normally distributed potential phenotypes, *z*, of all *N* individuals sampled from a population, both ancestors and descendant (plus hybrids, if included). Also consider vector **w**, which is the survivorship status of all individuals at the time that *z* is expressed (all elements of **w** are 0 or 1). Their joint probability density is

(3) p(z,w|d,m)=p(z|a,d)p(w|a,m)

where the vector **d** contains parameters for the differences across generations, **m** the parameters for the survival process, and **a** the breeding values for *z*. Thus, the combined model includes a conditional model for the data, *p*(**z**|**a**,** d**), generated from the animal model of quantitative genetics (Lynch & Walsh 1998; Wilson et al., 2010), and a conditional model for missingness, *p*(**w**|**a**,** m**). This model structure implies that the association between the trait of interest and the failure to revive is induced by additive genetic effects (Steinsland et al., 2014). An additional parameter is estimated by this procedure that describes the association between the two conditional models; if the association is 0, the two models are unrelated and the invisible fraction ignorable. Using this approach, Steinsland et al. (2014) showed that selection on the breeding value for spot size in female barn owls (*Tyto alba*) occurs during the early nesting stage, even before the spots are fully formed.

This sort of analysis can be applied to a resurrection experiment if the relatedness among the stored propagules is known. This condition is met in the Project Baseline collection because the stored seeds are packaged by maternal sibships (although paternity is not known). Germination failure during the refresher generation can be recorded, and information on all subsequently expressed traits for the failed seeds entered as missing values in the data set. An SPM model may then be used to estimate the level of divergence between ancestor and descendant samples (and their hybrids) due to additive genetic variance, which quantifies the evolutionary response to selection exerted by global change.

## 4 DISCUSSION

A crucial assumption in resurrection experiments is that the resurrected individuals are a random sample of genotypes from the ancestral generation (Bennington & McGraw, 1995). If genotype influences survival through the storage and revival process, the phenotypic mean estimated among the survivors can deviate from the mean expected for the ancestral generation as a whole. If so, the baseline for estimating evolutionary response will be biased, potentially leading to a false inference. When selection exerted during storage goes in the opposite direction as selection in the wild, a true evolutionary response could go undetected—a false negative. False positives arise if no selection occurs in the wild, but selection during storage shifts the baseline. Minor biases may not affect qualitative inferences, but will lead to over/under estimates of evolutionary rate.

The calculations presented above indicate that under a worst‐case scenario, the estimated phenotypic mean for a late‐life trait can be highly biased by a strong genetic correlation to seed storage tolerance. However, when seed mortality is related to additional, uncorrelated factors, and/or when the genetic correlation to storage tolerance is weak, the bias is weak. Under some reasonable assumptions, estimates of the baseline mean will be off by less than 0.1 *SD*, even when seed mortality is high. A genetic correlation between storage survival and the trait mean signals that the invisible fraction of the sample cannot be ignored when estimating the ancestral phenotypic mean.

Methods to identify and potentially correct an invisible fraction bias depend upon genealogical information. Resurrection experiments have addressed evolutionary change in a variety of ecologically important traits (see Franks et al., 2017), but in most cases, the genealogical structure of the samples was unknown and perhaps was unknowable. However, it would be wrong, for several reasons, to conclude that such experiments are necessarily invalid. First, if revival rate of dormant propagules is high, bias will be trivial. Second, if the performance of ancestral and descendant generations is tested both in ancestral‐like and in descendant‐like environments, and if each does better in its own environment, adaptive phenotypic evolution over time is the parsimonious explanation (see Franks et al., 2017).

It is also parsimonious to conclude adaptation when focal traits simply have no functional relationship to processes that would influence propagule survival and emergence. Resistance to cyanobacteria toxins in *Daphnia* (Hairston et al., 1999) and herbicide resistance in morning glories (Kuester, Wilson, Chang, & Baucom, 2016) seem unlikely to have mechanisms in common with prolonged propagule survival. Comparative studies of contemporary populations can be used to detect genetically mediated functional relationships, although care must be taken to distinguish genetic correlation due to pleiotropy from that due to population structure (linkage disequilibrium). But, genes governing basic metabolism are likely to be expressed both during revival and subsequent growth. Alleles at metabolic loci that are mildly deleterious under typical conditions could be lethal for embryos under the stress of prolonged storage and revival.

Pleiotropic effects of loci expressed during both early and late stages are one source of an invisible fraction problem, but studies on germination rates in crop cultivars suggest a second path—genetically mediated maternal effects. Crop varieties with larger seeds commonly have higher germination rates, especially under stressful conditions such as high temperature or saline soils (Krishnasamy & Seshu,^1989^; Almodares, Hadi, & Dosti, 2007; Moud & Maghsoudi, 2008). These varieties also have faster growing seedlings. Although varietal differences in survival and subsequent growth could be due to with‐individual pleiotropy, they also could reflect transgenerational pleiotropy caused by loci that govern both growth and maternal provisioning. An allele that increases the pool of maternal resources for seed production, or one that influences the number of seeds drawing upon that pool, would also influence offspring size and, hence, survival. As that offspring germinates and develops, it will express the alleles inherited from the mother. Offspring produced by other mothers, with weaker alleles, do not survive to express the focal trait. In such a case, supporting studies on the covariance between seed size and the focal trait could be useful in correcting for an invisible fraction bias.

Fruiting phenology in plants presents another example of a genetically mediated maternal effect that could produce an invisible fraction problem. If seeds are collected too early in the season, those from late‐fruiting plants will not have accumulated their full complement of cryoprotectants (Walters, 2015). When stored under the conditions used for the Project Baseline collection, seeds carrying genes for late‐flowering could have lower storage survival. Fortunately, the Project Baseline sampling protocol was designed to alleviate this and related problems by making repeated collections encompassing the phenological variation in target populations (Etterson et al., 2016; Franks et al., 2017).

Although low germination does not lead inevitably to a strong bias, high germination precludes it. Some studies using the resurrection approach have reported similar germination rates in the ancestral and descendant generations (Franks et al., 2007; Kuester, Chang, & Baucom, 2015; Thomann et al., 2015). Based on this similarity, investigators (including myself) have made the tacit assumption that germination failures have generated data that are MCAR or MAR. However, data can be MNAR even with equal germination rates in the ancestral and descendant samples (Figure 1c). To make the most of resurrection experiments, both samples must have known genealogical structure. No doubt, advances in statistical methodology will continue to refine ways to use information on relatedness to account for bias in estimates of evolutionary response in natural populations. When the final release of Project Baseline seed becomes available, *ca*. year 2065, future investigators will be able to use family structure to estimate the range of potential bias.
