# Supplementary material for: Detecting the “invisible fraction” bias in resurrection experiments
Source: Evol Appl. 2017 Sep 23;11(1):88–95. doi: 10.1111/eva.12533 (PMC5748523; doi:10.1111/eva.12533)
Supplement: Supplementary file 1 [file EVA-11-88-s001.docx]

**SUPPLENTAL MATERIAL –R-code for simulations**

############################################

### Simulated data for PROJECT BASELINE

### and the 'invisible fraction' paper

############################################

library(MASS)

library(doBy)

output.table <-table(NA, nrow=1, ncol=9)

############################################

# SET UP SMAPLE SIZES AND VARIANCES

############################################

G<-200 #Number of maternal half sipships

n<-20 #Number of seeds per sibshi;

gen.cor<-0.95 #gen3tic correlation bewteen traits y and z

env.cor<-0.95 #Environmental correlation between y and z

N<-G*n #total smaole size of seeds

k<-10 #Stringency of the relationship between y and seed survivorship

############################################

# CREATE CORRELATED GENETIC VALUES

############################################

for (i in 1:100)

{

g.focal<-c(1,gen.cor) #row 1, gen cor marix

g.seed<-c(gen.cor,1) #row 2, gen cor marix

G.COR<-rbind(g.focal,g.seed) #genetic correlation matrix

G.V<-c(0,0)

G.values<-mvrnorm(G,mu=G.V,Sigma=G.COR) #Generatie breeding vaues for y and z

############################################

#CREATE ENVIRONMENTAL DEVIATIONS

############################################

e.focal<-c(1,env.cor)

e.seed<-c(env.cor,1)

E.COR<-rbind(e.focal,e.seed)

E.V<-c(0,0)

E.values<-mvrnorm(N,mu=E.V,Sigma=E.COR) #Generate environmental deviations for y and z

############################################

#BUILD SIMULATED DATA SET

############################################

fam<-as.factor(rep(1:G, each=n)) #Generate column of family ID numbers

g.f<-rep(G.values[,1], each=n) #Replicate family mean for focal trait, n-times per family

e.f<-E.values[,1] # environemntal devialtions for focal trait

focal.t<-scale(g.f+e.f ) #Phenotypic Value of focal trat, for each sib, each family

g.s<-rep(G.values[,2], each=n) #Same, for the seed trait

e.s<-E.values[,2] #Same, for the seed trait

seed.t<-scale(g.s+e.s) #Same, for the seed trait

sim.data<-data.frame(fam,focal.t,seed.t) #Data Frame:family ID, focal trait, seed trait

############################################

p<-(1-(1/(1+exp(k*seed.t)+0))) #Fitness function for Seed Trait

fit<-rbinom(n=nrow(sim.data),1,prob=p) #survival of individual 'i'

sim.data.x<-cbind(sim.data,fit) #add survival to data set

sim.data.fit<-sim.data.x[which(fit==1),] #sub set data to include suvivors only

means.all<-summaryBy(seed.t+focal.t+fit~fam, data=sim.data.x,FUN=mean)

means.surv<-summaryBy(seed.t+focal.t+fit~fam, data=sim.data.fit,FUN=mean)

var.seed.t<-var(means.all[,2])

rel.fit<-fit/mean(sim.data.x[,4])

sel.grad.data<-cbind(sim.data.x[,2],rel.fit)

sel.grad.matrix<-cov(sel.grad.data)

gen.cor.matrix<-cor(means.all[,2:4])

################################################

mort<- mean(1-means.all[,4]) #proportion of seed that fail to revive

bias.mean<-mean(sim.data.fit[,2]) #mean of focal trat among survivors (=0, if no bias)

bias.fam.means<-mean(means.surv[,3])

focal.mort.cor<-gen.cor.matrix[2,3]

sel.grad.seed.t<-sel.grad.matrix[1,2]

output.row<-cbind(G,n,k, gen.cor,mort,bias.mean,bias.fam.means, focal.mort.cor, sel.grad.seed.t)

output.table<-rbind(output.table,output.row)

}

RRR<-as.data.frame(output.table) #initialze output tables

CCC<-as.factor(RRR$gen.cor)

KKK<-as.factor(RRR$k)

RRR<-as.data.frame(cbind(RRR,CCC,KKK))

is.data.frame(RRR)

#######################################################################

write.table(RRR,file = "output.mort.50.txt", col.names = T, quote = F)

**SUPPLENTAL MATERIAL –Figure S1**


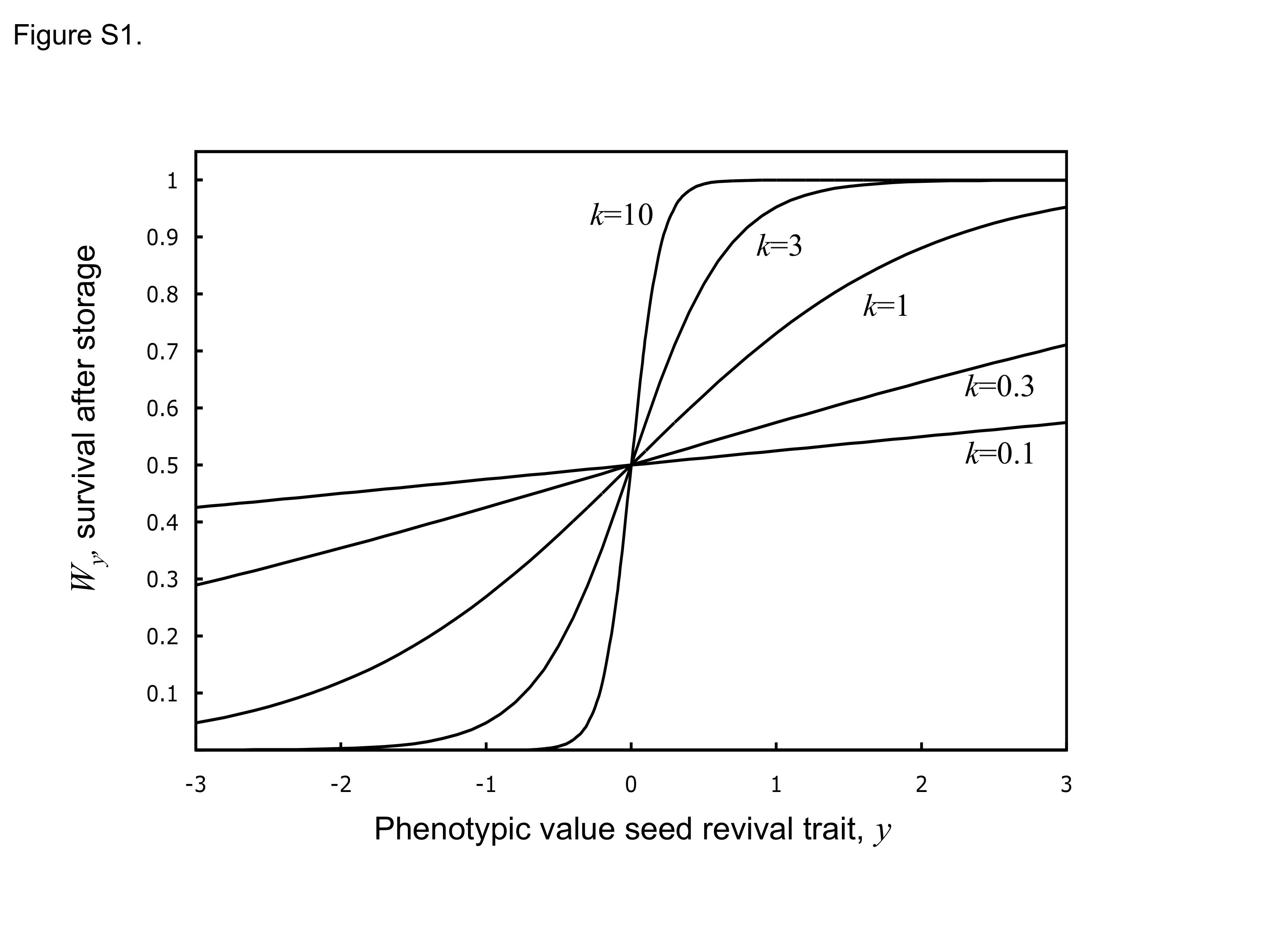


Fitness function of seed trait y. As *k* increases, the function approaches truncation selection, whereas selection is weak and the function approaches linearity when *k* is small. In all instance, 50% of all seed survives.
